# Supplementary material for: Hierarchical Nickel Cobalt Phosphide @ Carbon Nanofibers Composite Microspheres: Ultrahigh Energy Densities of Electrodes for Supercapacitors
Source: Nanomaterials (Basel). 2023 Nov 10;13(22):2927. doi: 10.3390/nano13222927 (PMC10675319; doi:10.3390/nano13222927)
Supplement: Supplementary file 1 [file nanomaterials-13-02927-s001.zip › nanomaterials-2673914-supplementary.pdf]

Supporting Information

Hierarchical Nickel Cobalt Phosphide @ Carbon Nanofibers Composite Microspheres: Ultrahigh Energy Densities of Electrodes for Supercapacitors

Jinqiao Zhang, Meiling Cen, Tao Wei, Qianyun Wang and Jing Xu *

College of Materials and Metallurgy, Guizhou University, Guiyang 550025, China
* Correspondence: jxu8@gzu.edu.cn

Supporting Figures:

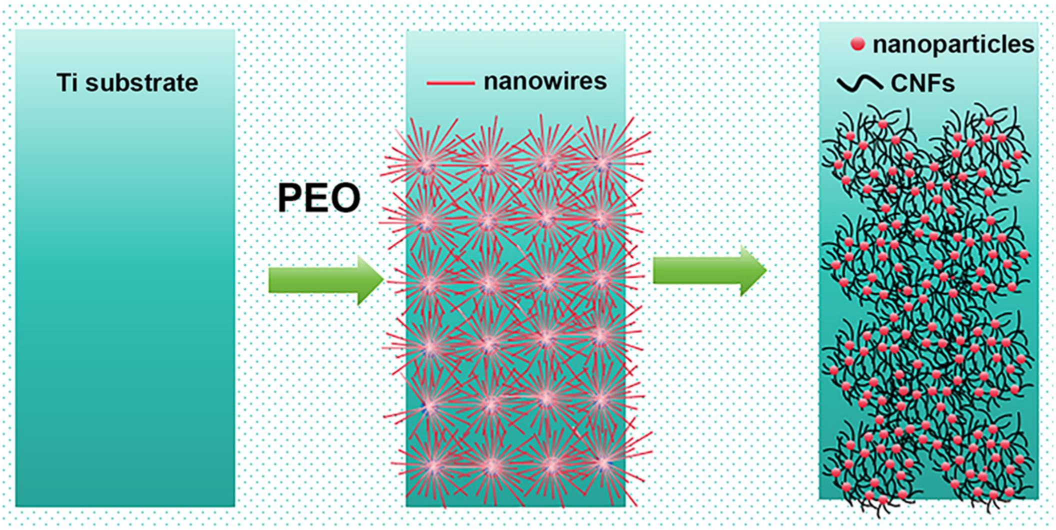

Figure S1. Schematic illustration of synthesis CoNiP@CNFs nanocomposites on a Ti substrate.

Table S1. Chemical composition of used electrolytes for PEO process.

Name	Chemical formula	Concentration (M)	Molecular weight (g/mol)
Sodium tetraborate	$Na_2B_4O_7 \cdot 10H_2O$	0.034	381.38
Trisodium phosphate	$Na_3PO_4 \cdot 12H_2O$	0.066	380.12
Sodium tungstate	$Na_2WO_4 \cdot 2H_2O$	0.006	329.85
Calcium acetate	$C_4H_6CaO_4 \cdot H_2O$	0.025	176.18
Nickel acetate	$C_4H_6NiO_4 \cdot 4H_2O$	0.06	248.84

Cobalt acetate	$C_4H_6CoO_4 \cdot 4H_2O$	0.04	249.08

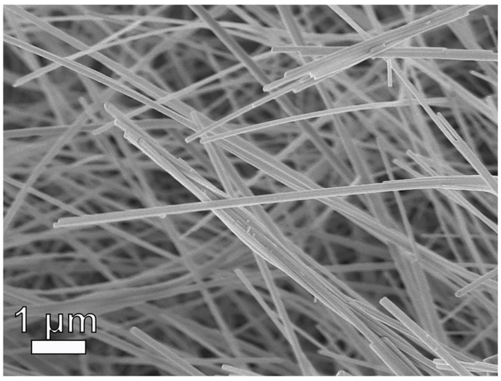

Figure S2. SEM image of Ni_5TiO_7 nanowires.

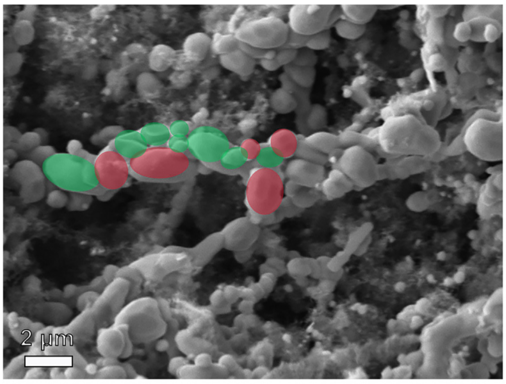

Figure S3. SEM image of $(Ni_{1-x}Co_x)TiO_7$ nanowires subjected to CVD for 10 minutes in C_2H_2 atmosphere.

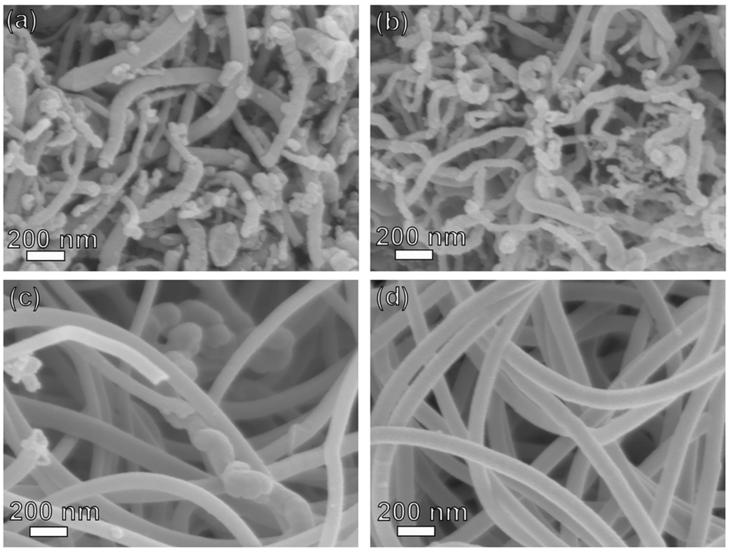

Figure S4. SEM images of a $(Ni_{1-x}Co_x)TiO_7$ film after the TCVD treatment at 700 °C in C_2H_2 atmosphere of (a) 3.75 torr, (b) 7.5 torr, (c) 37.5 torr and (d) 75 torr for 90 min.

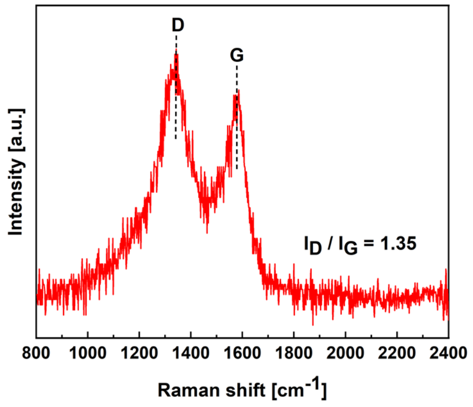

Figure S5. Raman spectroscopy of NiP@CNFs nanocomposites.

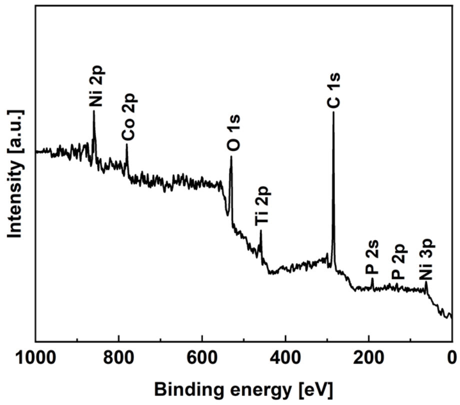

Figure S6. XPS survey spectrum of the CoNiP@CNFs nanocomposites.

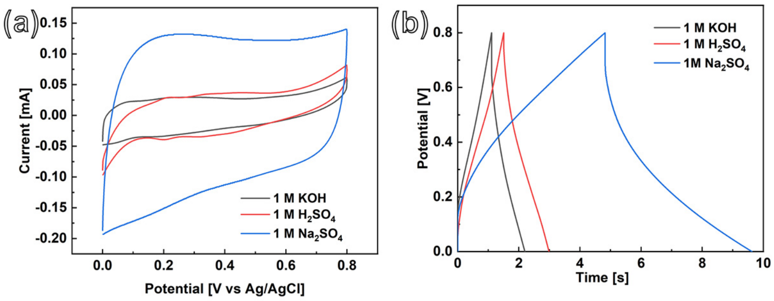

Figure S7. (a) CV and (b) GCD curves of CoNiP@CNFs nanocomposites in different electrolytes.

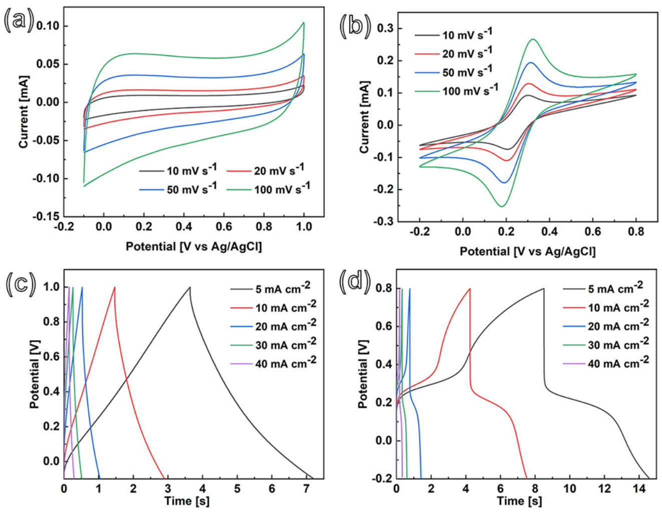

Figure S8. (a, b) CV and (c, d) GCD curves in (a, c) 1.0 M Na_2SO_4 and (b, d) 0.05 M Fe $(CN)_6^{3-/4-}$ + 1.0 M Na_2SO_4 electrolyte solution of NiP@CNFs nanocomposites.

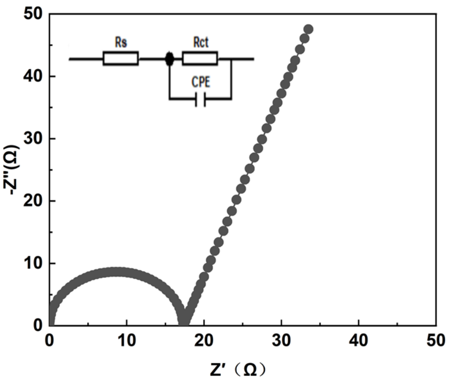

Figure S9. The EIS curve of NiP@CNFs nanocomposites.

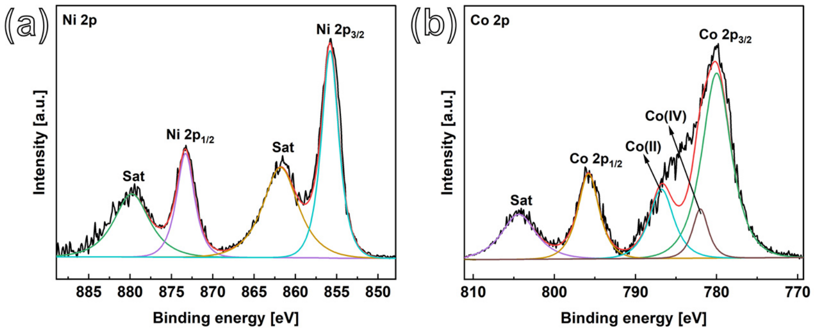

Figure S10. XPS spectra of (c) Ni 2*p*, (b) Co 2*p* core levels of CoNiP@CNFs composite after cycle life test.

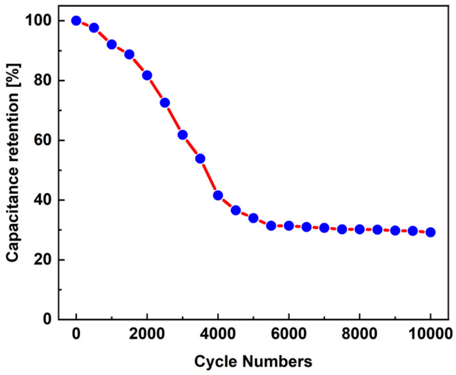

Figure S11. Capacitance retention of a CoNiP based (without growth of CNFs) electrode in 1.0 M Na_2SO_4 solution.

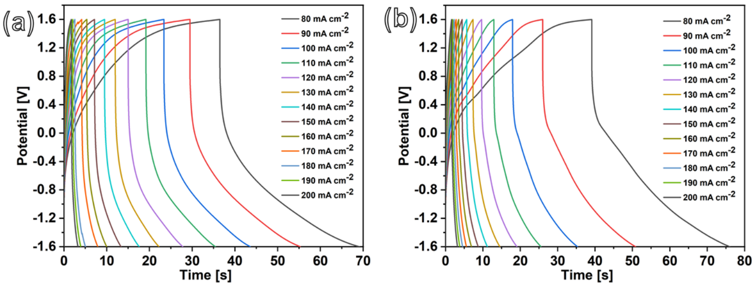

Figure S12. GCD curves of CoNiP@CNFs in (a) 1.0 M Na_2SO_4 and (b) 1.0 M Na_2SO_4 + 0.05 M $Fe(CN)_6^{3-/4-}$ solution at various current densities from 80 to 200 mA cm^{-2}.

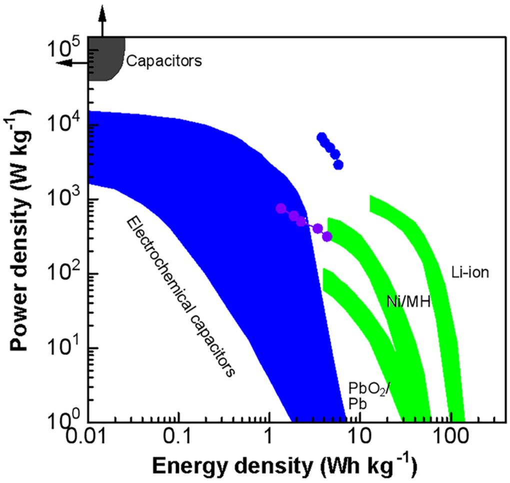

Figure S13. Comparison of Ragone plots of the NiP@CNFs based EDLCs in 1.0 M Na_2SO_4 (purple dotted line) and based PCs in 0.05 M Fe $(CN)_6^{3-/4-}$ + 1.0 Na_2SO_4 (blue dotted line) with those of traditional capacitors, ECs and batteries [S1]

References

S1. Simon, P.; Gogotsi, Y. Materials for electrochemical capacitors. *Nat. Mater* **2008**. 7, 845-854.
